# Time‐Resolved Intrinsic and Extrinsic Photoresponse of Colloidal Short‐Wavelength Infrared‐Active Indium Antimonide Quantum Dot Photodetectors

**DOI:** 10.1002/smsc.202500276

**Published:** 2025-07-22

**Authors:** Yongju Kwon, Zhouxiaosong Zeng, Fabian Strauß, Eric Juriatti, Patrick Michel, Heiko Peisert, Marcus Scheele

**Affiliations:** ^1^ Institute of Physical and Theoretical Chemistry University of Tübingen Auf der Morgenstelle 18 72076 Tübingen Germany

**Keywords:** III–V semiconductor nanocrystals, asynchronous optical sampling, colloidal quantum dots, indium antimonide, photodetectors, photoresponse time, short‐wavelength infrared

## Abstract

Colloidal indium antimonide (InSb) quantum dots (QDs) are highly promising nanomaterials for short‐wavelength infrared (SWIR) photodetectors due to their optical properties, solution processability, and low toxicity. Here, surface engineering of colloidal InSb QDs and an investigation of the intrinsic and extrinsic photoresponse time (*τ*_In_ and *t*_Ex_) of InSb QD photodetectors is presented. Chloride (Cl^−^) ligands are chosen for surface engineering and their effect is studied by X‐ray photoelectron spectroscopy. Using a pump‐probe technique based on asynchronous optical sampling (ASOPS), we find that *τ*_In_ of Cl‐capped InSb QDs (InSb‐Cl QDs) can be described by two components of 1.5 ns and 200 ps. By studying the dependence of these components on the voltage, the excitation power, and the temperature, we assign them to trap‐assisted Auger recombination and carrier trapping. For *t*_Ex_, much faster rise times (9.77 μs) than fall times (635 μs) indicate prolonged recovery due to slow release of trapped carriers is obtained. We devise measures to partially mitigate this drawback, enabling submicrosecond photo switching and a 3 dB bandwidth of 5 MHz. These findings highlight the potential of environmentally benign and high‐speed SWIR photodetectors based on colloidal III‐V semiconductor nanomaterials.

## Graphical abstract

The authors report chlorine (Cl) surface engineering of colloidal indium antimonide (InSb) quantum dots (QDs), which are active at 1560 nm, and an investigation of the intrinsic and extrinsic photoresponse time (*τ*_In_ and *t*_Ex_) of InSb‐Cl QD photodetectors. Asynchronous optical sampling (ASOPS) is demonstrated to study *τ*_In_. Additionally, *t*_Ex_ and factors that limit the *t*_Ex_ of InSb‐Cl QD photodetectors are comprehensively analyzed.

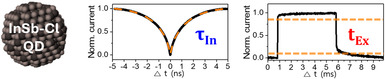

## 1 Introduction

Short‐wavelength infrared (SWIR) light penetrates harsh environmental conditions like nighttime, fog, and dust more effectively than visible light and produces higher‐resolution images compared to mid‐wave infrared (MWIR) or long‐wave infrared (LWIR).^[^ ^1^ ^]^ Consequently, SWIR photodetectors are increasingly in demand for applications such as night vision, biomedical imaging, environmental monitoring, and optical communication.^[^ ^2^ ^]^ Traditionally, bulk single crystalline materials such as InGaAs, InSb, InAsSb, and HgCdTe have been used for SWIR photodetectors. However, colloidal quantum dots (QDs) offer distinct advantages, including tunable absorption/emission spectra, cost‐efficient/scalable synthesis, solution processability, and compatibility with flexible substrates by simple deposition techniques such as spray coating, spin coating, etc.^[^ ^3^, ^4^ ^]^ Among SWIR‐active colloidal semiconductor QDs, indium antimonide (InSb) QDs are emerging as a highly promising nanomaterial due to a low‐toxicity alternative to Pb‐ or Hg‐based chalcogenide QDs.^[^ ^5^ ^]^ Bulk InSb features a narrow direct band gap of 0.17 eV at 300 K, high electron mobility (7.7 × 10^4^ cm^2^ Vs^−1^), low thermal conductivity (0.18 W cmK^−1^), and a large exciton Bohr radius (≈60 nm), allowing significant quantum confinement and size‐tuning of band gaps across the SWIR range.^[^ ^6^ ^]^

In 2012, Liu et al. introduced the first colloidal InSb QD photodetectors using S‐capped InSb QDs in a phototransistor architecture that absorbed SWIR light at 1400 nm.^[^ ^7^ ^]^ These QDs exhibited ambipolar charge transport, with electron and hole mobilities of 1.5 × 10^−4^ and 6 × 10^−4^ cm^2^ Vs^−1^, respectively, which are comparable to other SWIR‐active QDs like PbS or HgTe.^[^ ^8^ ^]^ Recently, in 2023, Shirahata's group advanced this work by developing the first photodiode with Br‐capped InSb QDs. By incorporating a ZnO electron transport layer and a PEDOT:PSS hole transport layer, they achieved a responsivity of 98 mA W^−1^ and extrinsic photoresponse times (*t*_Ex_) of 550 ms (*t*_Ex_rise_, rise time of *t*_Ex_) and 800 ms (*t*_Ex_fall_, fall time of *t*_Ex_).^[^ ^9^ ^]^ In their follow‐up study, S‐capped InSb QDs photodiodes exhibited an improved performance with an increased external quantum efficiency (EQE) and a reduction of *t*_Ex_ to 200 ms.^[^ ^10^ ^]^ This improvement was attributed to reduced ligand doping, which lowered trap states and dark currents. The Sargent group developed a halide‐capped InSb QD photodiode, achieving a high EQE of ≈75% at 1200 nm under −1 V, a detectivity of 10^11^ Jones, and *t*_Ex_rise_ of 7.3 μs as well as *t*_Ex_fall_ of 5.4 μs.^[^ ^11^ ^]^ The spectral sensitivity of the device was extended to 1400 nm, with an EQE of 33% and a detectivity of 10^12^ Jones.^[^ ^12^ ^]^ The high efficiency of the InSb QD photodiodes was attributed to a narrowed size distribution of the QDs and optimized surface engineering. Additionally, core/shell InSb/InP and InSb/InAs QD photodiodes have demonstrated enhanced stability, with InSb/InP QD devices showing strong durability over two months of ambient exposure without encapsulation.^[^ ^13^, ^14^ ^]^

Despite the recent progress in colloidal InSb QD photodetectors, which have improved EQE, detectivity, and stability,^[^ ^15^ ^]^ their photoresponse time, particularly the intrinsic response time (*τ*_In_) which is known to represent an upper limit to the speed of a device,^[^ ^16^ ^]^ remains underexplored (Table S1, Supporting Information). Additionally, the photodetection wavelengths of the reported colloidal InSb QD photodetectors are mostly restricted to below 1400 nm, because the synthesis of larger QDs with less quantum confinement has proven to be challenging. In telecommunication, the (C‐band) wavelengths around 1560 nm are ideal due to the exceptionally low transmission loss of 0.2 dB/km in silica optical fibers, coupled with minimal solar irradiance and reduced Rayleigh scattering.^[^ ^17^ ^]^ Recent advances in colloidal InSb QD syntheses, enabled by novel precursors, have allowed for the extension of the first excitonic absorption wavelength of InSb QDs up to 1900 nm.^[^ ^5^ ^]^

Here, we present an investigation of the photoresponse times for chloride‐capped InSb (InSb‐Cl) QD photodetectors operating at 1560 nm. Two types of photoresponse time are considered: i) the intrinsic response time, *τ*_In_, which is solely dependent on the material's characteristics and is typically measured using pump‐probe techniques;^[^ ^16^, ^18^ ^]^ ii) the extrinsic response time, *t*_Ex_, which reflects the overall device speed, such that it is not only influenced by the intrinsic material properties but also by external factors such as parasitic capacitances, the device geometry, and the resistance‐capacitance (RC) time.^[^ ^19^ ^]^

To explore *τ*_In_ of InSb‐Cl QD photodetectors, we perform pump‐probe pulse coincidence photoresponse measurements via asynchronous optical sampling (ASOPS). Unlike conventional pump‐probe setups, where pump and probe beams share the same repetition rate, with a controlled time delay via an optical delay line, ASOPS utilizes two electronically synchronized femtosecond fiber lasers with a slight difference in repetition rates.^[^ ^16^, ^20^ ^]^ This configuration creates continuously varying time delays (Δ*t*) between the pump and probe pulses (Scheme S1 and details in Supporting Information). Using this technique, we comprehensively characterized *τ*_In_ of InSb‐Cl QD photodetectors as a function of voltage, laser power, and temperature, providing insights into the underlying carrier dynamics. Additionally, *t*_Ex_ of InSb‐Cl QD photodetectors and factors limiting the speed of the devices are systematically studied.

## 2 Results and Discussion

### 2.1 Synthesis and Surface Engineering of SWIR‐Active InSb QDs

**Figure 1:**
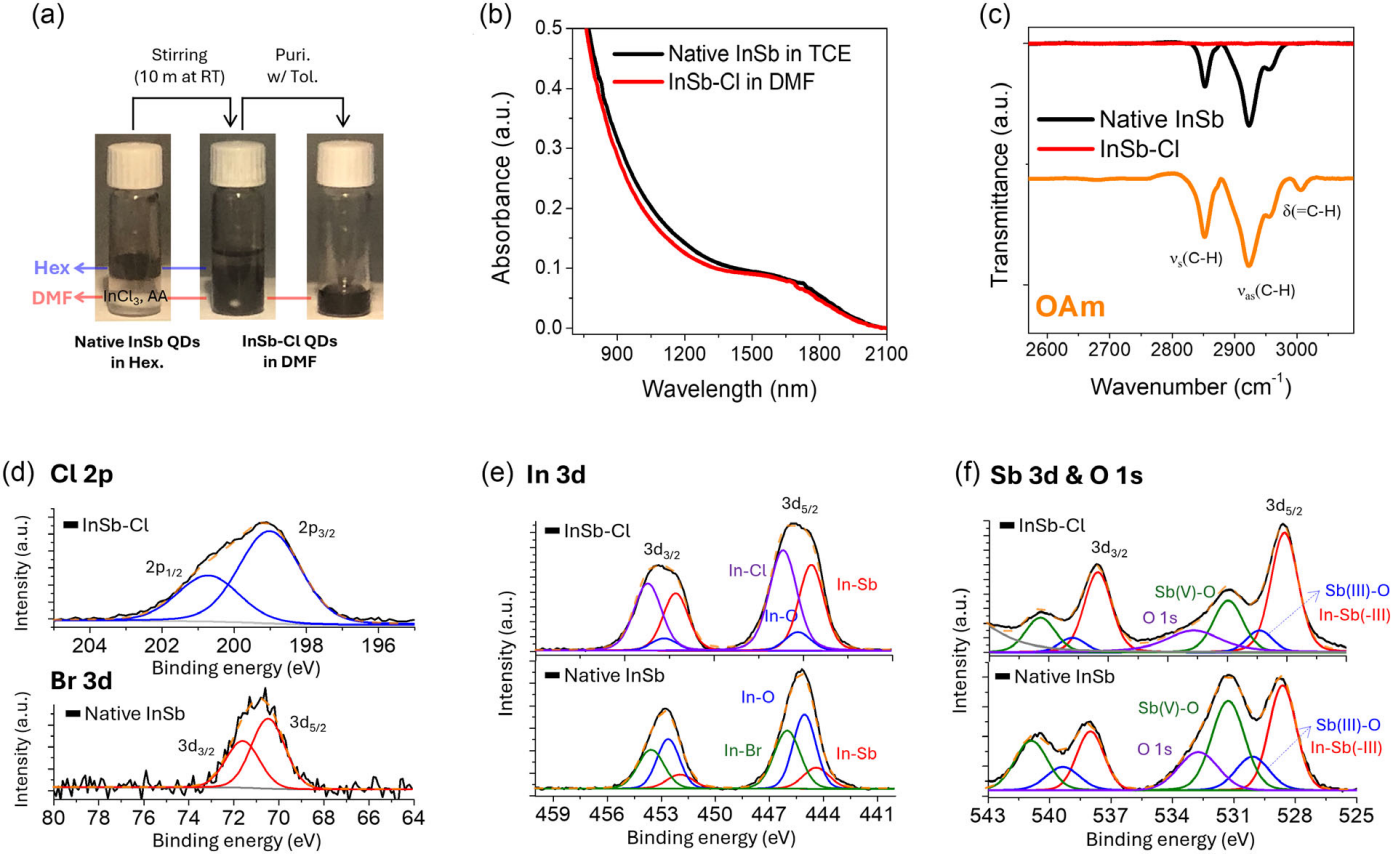
a) Photographs illustrating the two‐phase ligand exchange from native InSb QDs in hexane to InSb‐Cl QDs in DMF, b) UV–vis–NIR absorption, and c) FTIR spectra of InSb QDs before and after Cl^−^ ligand exchange. XPS spectra for d) Br 3d/Cl 2p, e) In 3d, and f) Sb 3d/O 1s of native InSb QDs and InSb‐Cl QDs.

Colloidal InSb QDs were synthesized following a previously reported protocol (See the supporting information (SI) for details).^[^ ^5^ ^]^ The as‐synthesized InSb QDs exhibit a first excitonic absorption peak around 1600 nm (Figure S1, Supporting Information), showing their suitability for SWIR photodetection. As confirmed by energy dispersive X‐ray spectroscopy (EDX) and X‐ray photoelectron spectroscopy (XPS) (Table S2a, Supporting Information and **Figure** **1**), the native ligands on the InSb QDs are bromide (Br^−^) as well as oleylamine (OAm). The In‐Br bonds of InSb QDs are formed due to the synthetic precursor, In(I)Br, during synthesis with OAm solvent. The OAm ligands provide steric stabilization and dispersion in hydrophobic solvents. However, their long alkyl chains impede charge carrier transfer between QDs in photodetectors, acting as insulating barriers. Therefore, we exchanged these native ligands with chloride (Cl^−^) by adapting a previously published procedure based on indium(III) chloride (InCl_3_) and ammonium acetate (AA) (details in Supporting Information).^[^ ^21^ ^]^ InCl_3_ and AA were dissolved in dimethylformamide (DMF), while the native InSb QDs were dispersed in hexane, forming two distinct layers (Figure 1a). After stirring for 10 mins, a phase transfer of the InSb QDs occurred due to the Cl^−^ ligand exchange. The UV‐vis‐NIR absorption spectra obtained before and after the Cl^−^ ligand exchange showed no significant changes, such as scattering or large blue shifts, confirming no aggregation or substantial etching reactions during the ligand exchange process (Figure 1b). Fourier transform infrared (FT‐IR) spectra were obtained for OAm, native InSb QDs, and InSb‐Cl QDs. OAm exhibited three distinct peaks at 2852 cm^−1^, 2923 cm^−1^, and 3006 cm^−1^, corresponding to the symmetric and asymmetric C—H stretching modes, and the =C—H bond of OAm, respectively.^[^ ^22^ ^]^ In the InSb‐Cl QDs, the C‐H stretching peaks remained unchanged, but the =C—H peak at 3006 cm^−1^ disappeared, possibly due to a redox reaction during synthesis.^[^ ^5^ ^]^ For the InSb‐Cl QDs, the FT‐IR spectrum confirmed the removal of OAm ligands after ligand exchange, evident from the absence of the stretching C—H vibrations of OAm (Figure 1c).

To study the composition of the QDs before and after ligand exchange and to obtain a deeper understanding of their electronic structure, XPS measurements were performed since this technique allows a highly surface‐sensitive analysis of the material. By the investigation of the Br 3d‐ and Cl 2p‐ core levels, the presence of halides can be studied. The In 3d‐ and Sb 3d/O 1s core levels give valuable information about the oxidation states and binding situations of the metal atoms and are therefore beneficial for the elucidation of the chemical structure before and after ligand exchange.

*Halide core level spectra:* A Br 3d peak was observed exclusively before the Cl^−^ ligand exchange, while a Cl 2p peak appeared only afterward (Figure 1d, S2 and Table S3a, Supporting Information). While this is already strong evidence for a successful ligand exchange, we further confirm this result by EDX (Table S2b, Supporting Information). The apparent ease of Br^−^ replacement by Cl^−^ can be rationalized by the hard/soft acid‐base (HSAB) principle and the larger hardness of Cl^−^, making it a better match for hard In^3+^ ions. This is also reflected in the larger bond dissociation energies for In–Cl (439 kJ mol^−1^) compared to In–Br (418 kJ mol^−1^).^[^ ^23^ ^]^ Based on the halide XPS spectra with the FT‐IR data showing the absence of OAm on InSb‐Cl QDs, we conclude that all the OAm and Br ligands on native InSb QDs have been exchanged for Cl ligands.

*Indium 3 d‐core level spectra:* The In 3d spectrum of native InSb QDs can be described by three doublets (3d_5/2_ and 3d_3/2_ components), which can be assigned to In‐Sb at 444.4 eV and 452.0 eV; and In‐O at 445.1 eV and 452.6 eV, and In‐Br at 446.0 eV and 453.6 eV (Figure 1e and Table S3b, Supporting Information).^[^ ^5^, ^12^, ^24^ ^]^ The prominent In‐O peak relative to the In‐Sb peak indicates oxidation of the native InSb QDs, likely occurring due to short air exposure (5 mins) before XPS measurements. After the Cl ligand exchange, the InSb‐Cl QDs exhibit a strong In‐Cl peak at 446.1 eV and 453.7 eV for In 3d_5/2_ and In 3d_3/2_, alongside the prominent In‐Sb peak, signifying a high Cl^−^ binding ratio, which contributes to the high dispersibility of InSb‐Cl QDs in DMF. Also, the more pronounced In‐Sb peaks when compared to the In‐O peaks suggest reduced oxidation which can be attributed to the Cl^−^ ligands. Specifically, the In‐O component decreased from 44% in the native InSb QDs to 9% in the InSb‐Cl QDs (Table S3b, Supporting Information).

*Sb 3 d‐ and O 1s‐core level spectra:* We observed Sb(III)‐O and Sb(V)‐O peaks at 530.1 eV and 539.4 eV, and 531.30 eV and 540.9 eV, for Sb 3d_5/2_ and Sb 3d_3/2_, respectively, alongside In‐Sb(‐III) peaks at 528.6 eV and 538.0 eV for Sb 3d_5/2_ and Sb 3d_3/2_ (Figure 1f and Table S3c, Supporting Information).^[^ ^12^, ^24^ ^]^ The prominent O 1s peaks at 532.7 eV indicate the presence of oxide species such as In_2_O_3_, Sb_2_O_3_, and Sb_2_O_5_ (Figure 1e,f). After Cl^−^ ligand exchange, the oxide peaks in the Sb 3d and O 1s spectra significantly diminished with the prominent In‐Sb(‐III) peaks. Similar to the In 3d spectra, the Sb(III)‐O and Sb(V)‐O components in the Sb 3 d spectra decreased from 17% and 45% to 11% and 29%, respectively (Table S3c, Supporting Information), reaffirming the persistence of InSb‐Cl QDs against air. Concerning the improved resistance to surface oxidation after Cl‐exchange, we assume that the sterically hindered OAm cannot effectively passivate the surface against air exposure, whereas the short Cl^−^ ligand can densely passivate the surface with strong binding.

In summary, colloidal InSb QDs with a first excitonic absorption peak near 1600 nm were synthesized, and their native ligands, OAm and Br^−^, were successfully exchanged to Cl^−^ without visible etching or particle aggregation. XPS, FT‐IR, and EDX analyses confirmed the complete Cl^−^ ligand exchange, as well as the full removal of organic components from the native InSb QDs. We found a large dispersibility of InSb‐Cl QDs in DMF which we attribute to high Cl^−^ surface coverage. Additionally, Cl^−^ ligands provide increased robustness toward air for InSb‐Cl QDs compared to native InSb QDs.

### 2.2 Time‐Resolved Photoresponse of InSb‐Cl QD Photodetectors

**Figure 2:**
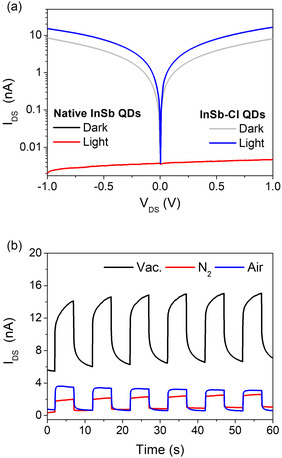
a) *I–V* curves of native InSb QD and InSb‐Cl QD photodetectors with or without light and b) ON/OFF photoresponse characteristics of InSb‐Cl QD photodetectors under vacuum, N_2_, or air using a square pulse laser (*V*_DS_, drain‐source voltage: 1 V, *λ*_Exc_., excitation wavelength of laser: 636 nm, *f*, laser frequency: 0.1 Hz, P, laser power: 55 μW).

While previous studies of InSb QD‐based photodetectors were mainly focused on the responsivity in the SWIR, here we specifically target their time‐resolved photoresponse. To this end, we fabricated lateral, two‐terminal photodetectors with native InSb QDs and InSb‐Cl QDs by spin‐coating on SiO_2_/Si substrates with Au/ITO electrodes (details in SI). Current‐voltage (I‐V) measurements under vacuum showed that the native InSb QD photodetectors exhibited no detectable photocurrent due to the insulating nature of long alkyl chain ligands (**Figure** **2**a). In contrast, InSb‐Cl QD photodetectors demonstrated a photocurrent of tens of nA at 1 V, indicating better electronic coupling provided by the Cl^−^ ligand shell. Comparative tests on the InSb‐Cl QD photodetector with a square pulse laser (*V*_DS_, drain‐source voltage: 1 V; *f*, laser frequency: 0.1 Hz; *λ*_Exc_., excitation wavelength of laser: 636 nm; P, laser power: 55 μW) showed that the highest photocurrent occurred under vacuum, while N_2_ and air exposure led to reduced photocurrent (Figure 2b, S3 and S4, Supporting Information). The reduced photocurrent under N_2_ may result from N_2_ adsorption on QD surfaces, possibly interacting with trap sites.^[^ ^25^ ^]^ Likewise, exposure to air likely led to surface oxidation of InSb‐Cl QDs, forming In_2_O_3,_ Sb_2_O_3_, and Sb_2_O_5_ (Figure 1e,f), which reduces the photocurrent.

In the following, we investigate the transient photocurrents in InSb‐Cl QD photodetectors in more detail by employing three complementary experiments: 1) pump‐probe pulse coincidence measurements at 1560 nm using ASOPS (Scheme S1, and details in Supporting Information),^[^ ^16^, ^26^ ^]^ 2) steady‐state photoexcitation using a 636 nm square pulse laser with 100 Hz or 10 kHz repetition rate, and 3) non‐steady state photoexcitation using a 636 nm impulse laser with 100 kHz repetition rate.

With the first experiment, we specifically probe the intrinsic photophysical properties of the InSb‐Cl QDs, while the latter two experiments provide information about the time‐resolved photodetection performance in actual devices.

#### 2.2.1 Intrinsic SWIR Photoresponse Time of InSb‐Cl QD Photodetectors

**Figure 3:**
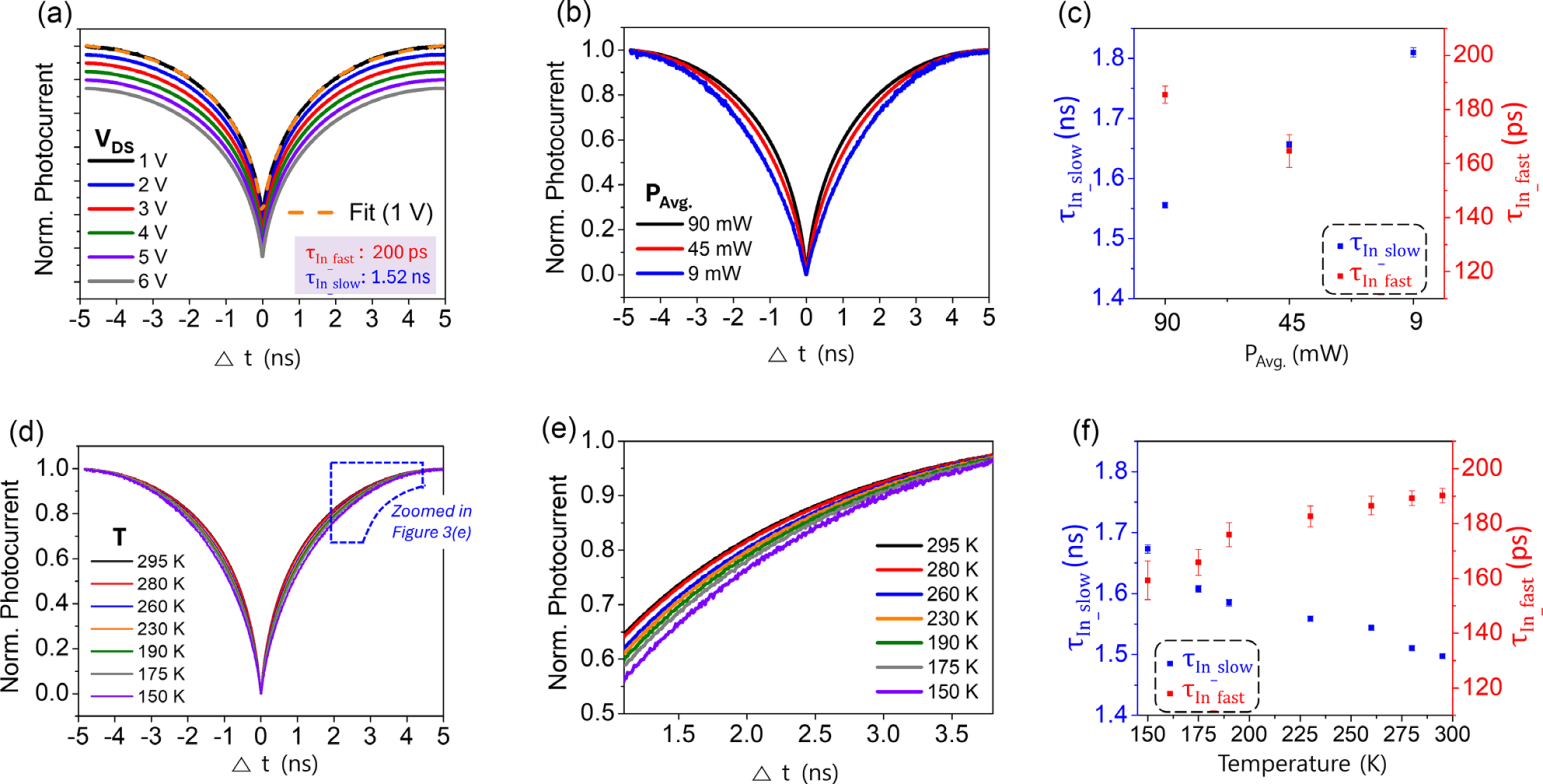
a) ASOPS signals of InSb‐Cl QD photodetectors measured under vacuum (*λ*_Exc_.: 1560 nm, *P*_Avg_., average power of the pump and probe lasers: 90 mW) at varying *V*_DS_ from 1 V to 6 V. The 1 V data is fitted with a second‐order exponential decay function (dotted yellow line). b) ASOPS signals demonstrate the effects of varying *P*_Avg_. A neutral density filter was applied to attenuate the power by 3 dB or 10 dB. c) *τ*_In_ values for slow (τ_In_slow_) and fast (*τ*_In_fast_) components, obtained by fitting the data in (b), are plotted as a function of P_Avg_. d) ASOPS data showing temperature variations, with e) a zoomed‐in view. f) *τ*_In_ for the slow (*τ*_In_slow_) and fast (*τ*_In_fast_) components, obtained by fitting the data in (d), are plotted as a function of temperature. The error bars in (c) and (f) represent the mean ± SD, with *n* = 2.

The ASOPS measurements, conducted under vacuum with a 1 V external bias, displayed a clear and symmetric ASOPS signal as a function of the pump‐probe delay time (**Figure** **3**a, black line), confirming the SWIR‐sensitivity of the QDs. The ASOPS data were fit to a second‐order exponential decay function (Figure 3a, dotted yellow line) with the two components *τ*_In_fast_ (fast component of *τ*_In_) = 200 ps and *τ*_In_slow_ (slow component of *τ*_In_) = 1.52 ns. The respective fitting coefficients of 0.189 and 0.848 (**Table** **1**) indicate the dominant role of the slow component. We suggest that these results may be rationalized in terms of photoexcited charge carriers that either transit through a thermally activated hopping mechanism^[^ ^27^ ^]^ or recombine with oppositely charged carriers (details in Supporting Information).^[^ ^16^ ^]^ To identify the most plausible mechanism, we analyzed the dependence of the two lifetime components on the voltage, the excitation power, and the temperature.

**Table 1:** The fitting parameters of *τ*_In_slow_ and *τ*_In_fast_ for InSb‐Cl QD photodetectors, measured at *V*_DS_ ranging from 1 V to 6 V. The values represent the mean (±SD), with *n* = 2. ASOPS data on both the right and left sides were fitted using the equation, y = A1 × *e* ^−*x*/*τ*1^ + A2 × *e* ^−*x*/*τ*2^ + y_0_. Here, *τ*1 and A1 represent *τ*_In_slow_ and its corresponding coefficient, while *τ*2 and A2 represent *τ*_In_fast_ and its coefficient.

	*V*_DS_ [V]	1	2	3	4	5	6
$\tau_{\text{In}\_\text{slow}}$	|A1|	0.848 (±0.002)	0.852 (±0.001)	0.854 (±0.001)	0.858 (±0.001)	0.858 (±0.001)	0.861 (±0.001)
	|τ1| (ns)	1.523 (±0.005)	1.506 (±0.004)	1.519 (±0.004)	1.529 (±0.004)	1.537 (±0.004)	1.541 (±0.004)
$\tau_{\text{In}\_\text{fast}}$	|A2|	0.189 (±0.002)	0.185 (±0.002)	0.185 (±0.002)	0.184 (±0.002)	0.183 (±0.002)	0.180 (±0.002)
	|τ2| (ps)	199 (±4)	193 (±3)	193 (±3)	192 (±3)	192 (±3)	191 (±3)
	y_0_	1.036 (±0.001)	1.037 (±0.000)	1.039 (±0.000)	1.010 (±0.000)	1.041 (±0.001)	1.042 (±0.001)

*Voltage dependence:* As detailed in Figure 3a, Table 1, and Figures S5–S7, Supporting Information varying the *V*_DS_ had no effect on the *τ*_In_ components, suggesting that the carrier dynamics are dominated by recombination, and not transit.

*Photoexcitation power dependence:* Neutral density filters were used to attenuate the average pump and probe laser power (*P*_Avg_) by 3 or 10 dB from the initial 90 mW. As the laser power decreased, the ASOPS signal broadened, indicating an overall slower *τ*_In_ (Figure 3b). Specifically, the *τ*_In_slow_ component increased from 1.56 ns to 1.81 ns under 9 mW (Figure 3c, Figure S8 and Table S4, Supporting Information). In contrast, *τ*_In_fast_ decreased from the initial 190 ps to 170 ps under 45 mW, and disappeared at 9 mW. This increase in *τ*_In_slow_ suggests that its origin lies in a trap‐assisted Auger (TAA) recombination via decoupled charge carrier decay.^[^ ^16^ ^]^ TAA recombination involves the recombination of electron‐hole pairs through trap states, transferring energy to a third carrier, which is subsequently excited to a higher energy level (Figure S9, Supporting Information). At sufficient photoexcitation power, the increased density of photoexcited charges within the QDs accelerates TAA recombination, leading to the shorter *τ*_In_slow_. In contrast, radiative recombination times for InSb QDs, typically ranging from tens to thousands of nanoseconds,^[^ ^7^, ^11^, ^12^, ^28^ ^]^ significantly differ from *τ*_In_slow_ (≈1.5 ns), supporting the non‐radiative recombination origin.

**Scheme 1:**
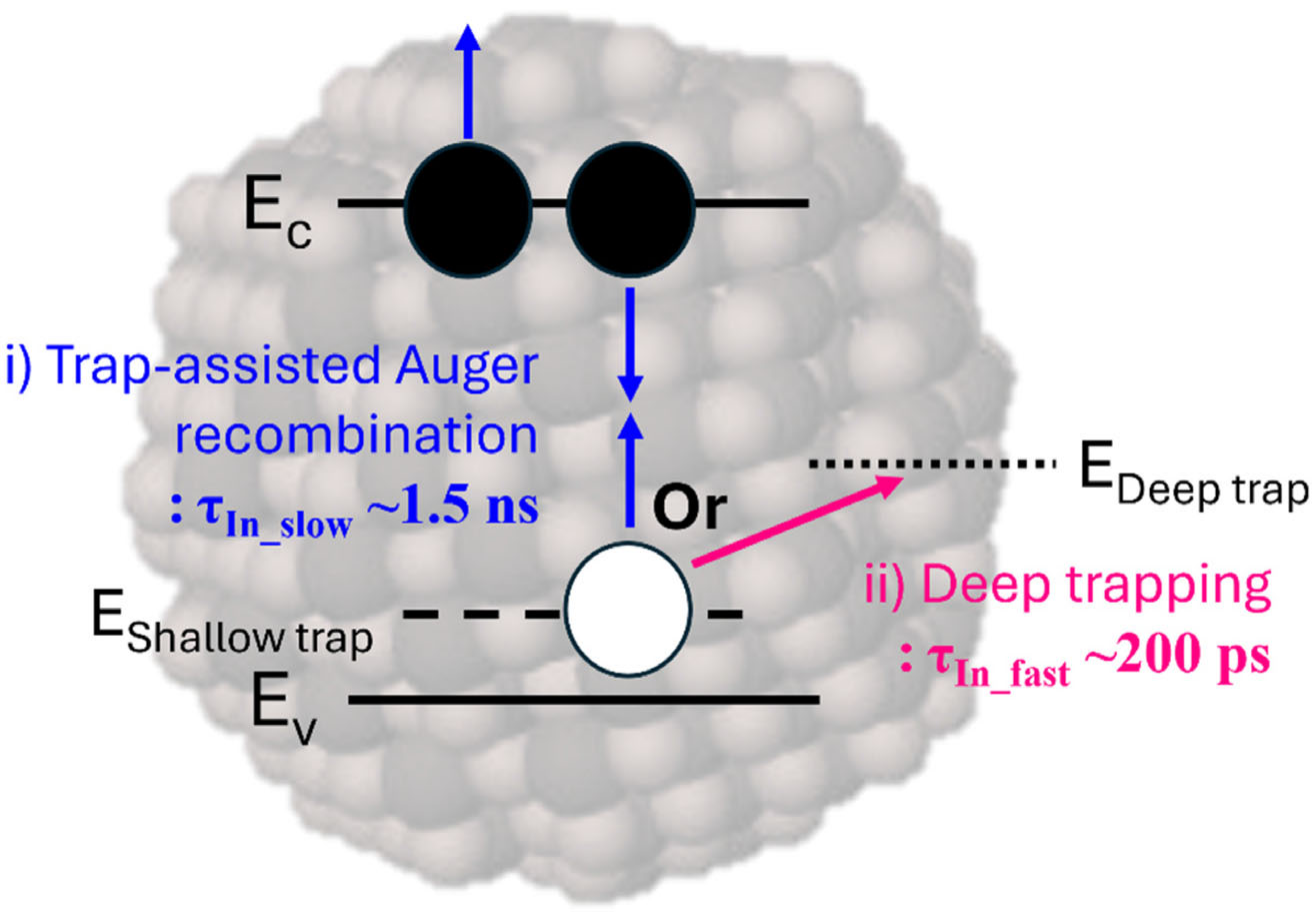
Schematic representation of charge carrier dynamics in InSb‐Cl QDs. A charge carrier at shallow traps undergoes TAA recombination within ≈1.5 ns or moves to deep trap sites within ≈200 ps.

In contrast, the *τ*_In_fast_ component decreased from 190 to 160 ps in the same temperature regime (Figure 3c and Table S4, Supporting Information). These picosecond‐scale values likely correspond to charge carrier trapping or multiple exciton‐related recombination.[^29^, ^30^] One way of understanding the diverging nature between *τ*_In_fast_ and *τ*_In_slow_ is through TAA at shallow traps in the case of *τ*_In_slow_ and transitions from shallow to deep traps for *τ*_In_fast_ (**Scheme** **1**). The slower TAA at shallow traps appears to facilitate the faster transition to deep trapping. We note that introducing halide ligands into InSb QDs has been shown to induce picosecond‐scale transient states, which were attributed to enhanced trap states.^[^ ^11^, ^13^, ^31^ ^]^

*Temperature dependence:* From 295 K to 150 K, the *τ*_In_slow_ component increased from 1.50 ns to 1.67 ns, implying the influence of charge carrier‐phonon interaction (Figure 3d–f, S10 and Table S5, Supporting Information). We argue that reduced phonon activity at lower temperatures slows the TAA process,^[^ ^29^ ^]^ leading to longer *τ*_In_slow_ values. The temperature‐dependent behavior highlights the interplay between phonons and charge carriers, possibly involving Auger recombination, which depends on Coulomb coupling between electrons and holes.^[^ ^32^ ^]^ When surface trapping decouples charge carriers, the decay instead proceeds via phonon interactions. In other words, the temperature‐dependent ASOPS data imply that TAA (represented by *τ*_In_slow_) likely originates from trap states. (Scheme 1).

#### 2.2.2 Extrinsic Photoresponse Time of InSb‐Cl QD Photodetectors

Having determined the upper‐speed limit of InSb‐Cl QD photodetectors from the intrinsic photoresponse measurements to be ≈1.5 ns, we now analyze the extrinsic response time, *t*_Ex_, and factors limiting the speed in real devices. This parameter is obtained by monitoring the photocurrent at the beginning of a pulse to increase from 10% to 90% of its final magnitude (*t*_Ex_rise_) or to decrease from 90% to 10% (*t*_Ex_fall_) at the end of a pulse.

**Figure 4:**
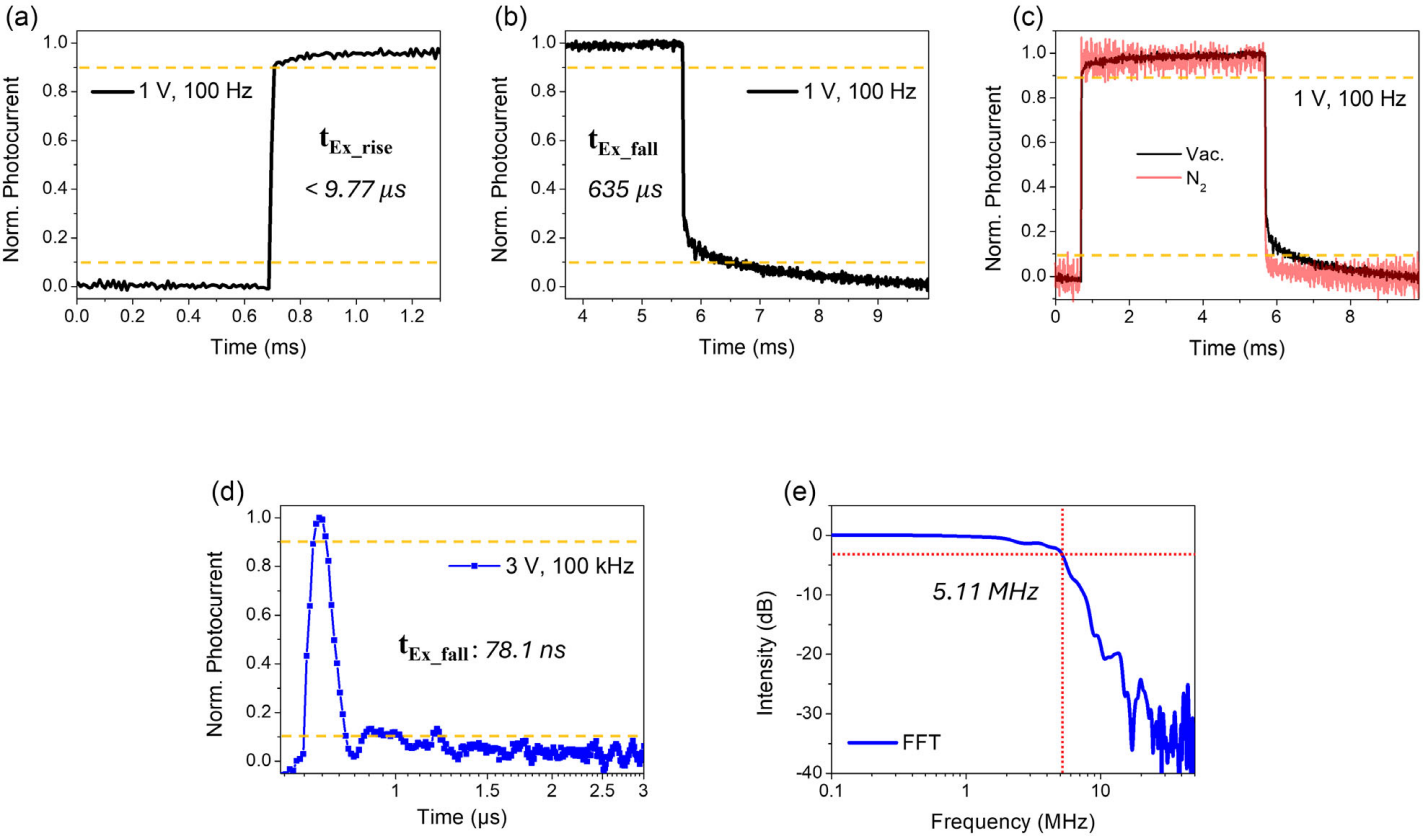
Photoresponse characteristics of InSb‐Cl QD photodetectors under a square pulse laser illumination (*f*: 100 Hz, *λ*_Exc_.: 636 nm, P: 2 mW). a) Rise time (*t*_Ex_rise_) and b) fall time (*t*_Ex_fall_) at 1 V under vacuum, and c) environment comparison between vacuum and N_2_. d) Photoresponse characteristics under impulse laser illumination (*f*: 100 kHz, *λ*_Exc_.: 636 nm, P: 28.5 μW) and e) the corresponding 3 dB bandwidth, derived from FFT of the power spectrum.

*Steady‐state measurements*: Using a square pulse laser (*V*_DS_: 1 V, *f*: 100 Hz, *λ*_Exc_.: 636 nm, P: 2 mW), we obtained *t*_Ex_rise_ = 9.77 μs and *t*_Ex_fall_ = 635 μs under vacuum (**Figure** **4**a,b). These microsecond‐scale *t*_Ex_ values are relatively fast given the photoconductor architecture, compared to the reported values for InSb QD photodiodes (Table S1, Supporting Information).^[^ ^9^, ^10^, ^11^, ^12^, ^13^, ^14^ ^]^ The significantly slower fall time compared to the rise time indicates a prolonged recovery time primarily caused by slow release of trapped carriers,^[29b]^ consistent with the ASOPS results (Figure 3 and Scheme 1). Under N_2_, *t*_Ex_fall_ decreased to 48.8 μs (Figure 4c), possibly due to the passivation of trap sites.

In general, *t*_Ex_ of a photodetector is subject to three main limitations, namely the carrier diffusion time, the carrier drift/transit time (*t*_Trans_), and the RC time (*t*_RC_) of the device. To determine for a specific device, which process dominates its response time, it is instructive to test for a dependence of *t*_Ex_ on the channel length and the applied bias, as well as calculating the RC constant. If *t*_Trans_ was the limiting factor for the speed of the device, increasing the channel would affect *t*_Ex_. However, no significant differences in *t*_Ex_ were observed between channel lengths of 2.5 μm and 5 μm (Figure S11, Supporting Information). In contrast, we observed a prolonged response time for larger *V*_SD_ (Figure S12, Supporting Information). This behavior can be interpreted as the recovery time of the device,^[29b]^ which most likely slows down at higher biases due to the delayed release of trapped carriers caused by the increased number of trapped carriers (Figure S13, Supporting Information). To estimate *t*_RC_, we conducted impedance measurements and found a capacitance on the order of 10 pF (Figure S14, Supporting Information). Multiplied with typical photoresistances of 100–200 MΩ (Figure S15, Supporting Information) and the factor 2.2 to account for the 10%–90% definition of *t*_Ex_, *t*_RC_ is on the order of 2–3 ms. This is in reasonable agreement with *t*_Ex_fall_ measured at 100 Hz under vacuum, indicating that the photodetectors are probably RC‐limited.

Considering the large difference between *t*_Ex_rise_ and *t*_Ex_fall_, the device can be measured at higher laser repetition rates (*f*: 10 kHz) to explore the temporal limit of *t*_Ex_rise_ (Figure S15, Supporting Information). In this process, the slow trap filling and releasing processes cannot take place anymore, and nearly 25% of the signal is lost. Thereby, the *t*_Ex_rise_ decreases to <1 μs, highlighting the potential for high‐speed photodetection.

*Non‐steady state measurements:* To gauge the potential of the InSb‐Cl QD photodetectors for actual telecommunication applications, we determined the electrical 3 dB bandwidth using an impulse laser (*V*_DS_: 3 V, *f*: 100 kHz, *λ*_Exc_.: 636 nm, P: 28.5 μW). Under these conditions, we found *t*_Ex_fall_ = 78.1 ns (Figure 4d), and from a Fourier transform of the corresponding power spectrum, we obtained the 3 dB bandwidth as 5.11 MHz (Figure 4e). Similar to the 10 kHz steady‐state measurements, the 100 kHz repetition rates used here will invoke some signal loss due to cutting out the slowest components, such as the release of trapped carriers. However, the remaining photocurrent is still large enough for stable operation at MHz frequencies.

## 3 Summary

We have fabricated SWIR‐active photodetectors based on InSb QDs with strong absorption in the third telecommunication window (1560 nm) and employed chloride surface functionalization to enhance charge carrier transfer and stability toward oxidation. We determined the intrinsic time‐resolved photoresponse of the QDs by ASOPS and found that it is most likely driven by TAA recombination, as well as carrier deep trapping. The ASOPS study represents the main advantage of this work, being the first report on the intrinsic photoresponse of InSb QD photodetectors. Additionally, our analysis of the extrinsic photoresponse revealed a much faster rise than fall times, indicating prolonged recovery due to the slow release of trapped carriers. This drawback can be overcome by operating at kHz repetition rates, which retains 75% of the photocurrent, decreases the rise time to <1 μs and enables a 3 dB bandwidth of 5 MHz. Future studies should focus on controlling trap states of InSb QDs and optimizing the device geometry to reduce the RC time of the photodetectors, since our intrinsic measurements suggest that nanosecond switching at 1560 nm should be possible with this material in principle.

## 4 Experimental Section

4.1

4.1.1

##### Chemicals

Indium(I) bromide (In(I)Br, 99.999%) and chloroform (99.9%, extra dry) were obtained from Thermo Scientific. Tris(dimethylamido)antimony (Sb(NMe_2_)_3_, >95%) was sourced from Gelest. Acros supplied octadecene (ODE, 90%), oleylamine (OAm, 80%–90%), toluene (99.85%, extra dry), hexane (97%, extra dry), acetone (99.8%, extra dry), N, N‐dimethyl formamide (DMF, 99.8%, extra dry), and tetrachloroethylene (TCE, 99+%, extra dry). Sigma‐Aldrich provided methyl acetate (99.7%, extra dry). Indium(III) chloride (InCl_3_, 99,99%) and tri‐n‐octylphosphine (TOP, 97%) were purchased from Abcr. Ammonium acetate (AA) was from Universität Tübingen. All chemicals were used without further purification.

##### Preparation of InSb QDs

*Synthesis.* The synthesis of InSb QDs was carried out following a previously reported method.^[^ ^5^ ^]^ First, 0.17 mmol of Sb(NMe_2_)_3_ was dispersed in 0.4 mL of degassed OAm at 50 °C in a glove box. The Sb(NMe_2_)_3_ was added to 1.0 mmol of In(I)Br in 1.6 mL of degassed OAm solution to prepare InSb precursor solution and they were stirred for 1 hr at 50 °C. In a three‐necked round‐bottom flask, 4 mL of ODE was degassed for 1 hr at 120 °C, then heated to 260 °C under Ar atmosphere. After that, 0.3 mL of TOP and 2 mL of InSb precursor solution were injected into the ODE solvent at 260 °C. The reaction mixture was kept at this temperature and stirred for 25 mins.

*Purification.* First, 0.5 mL of the crude sample was centrifuged at 4,000 rpm for 1 min to get a supernatant solution without metallic byproducts. The supernatant solution was mixed with 0.5 mL of anhydrous chloroform, 3 mL of anhydrous methyl acetate, and 3 mL of anhydrous acetone. The mixture solution was centrifuged at 4,000 rpm for 5 mins. Precipitants were dispersed in anhydrous TCE or hexane.

##### Ligand Exchange

*Cl ligand exchange.* The Cl^−^ ligand exchange method for InSb QDs was adapted from a previously published procedure.^[^ ^33^ ^]^ The purified InSb QDs (50 mg mL^−1^) in 0.5 mL of hexane were mixed with 0.125 mmol of InCl_3_ and 0.075 mmol AA in DMF solution, and the mixture was stirred for 10 mins. After discarding the hexane layer, the remaining 0.5 mL of InSb‐Cl QDs in DMF was mixed with 2 mL of toluene and then centrifuged at 4,000 rpm for 1 min. The precipitated InSb‐Cl QDs were redispersed in DMF. The InSb‐Cl QDs solution in DMF was filtered through a 0.2 μm PTFE membrane before the fabrication of InSb‐Cl QD photodetectors.

##### Photodetector Fabrication

An interdigitated electrode substrate, commercially obtained from Fraunhofer IPMS, was utilized to fabricate InSb‐Cl QD photodetectors. The source and drain pads comprised 10 nm ITO and 30 nm Au electrodes on a Si/SiO_2_ layer. The substrate was washed with acetone, ethanol, and isopropanol, followed by UV–ozone treatment (UB1101AR‐1 for UV power supply and PL16‐110B‐1 for photo surface processor from Universität Tübingen) for 10 mins. The QD solution was spin‐coated onto the substrate inside a glove box at 1200 rpm for 30 secs. The InSb‐Cl QDs solution in DMF had a concentration of 140 mg mL^−1^. The InSb‐Cl QD film was dried under vacuum for 1 hr before the optoelectronic measurements.

##### Characterization

UV–vis–NIR absorption spectra were recorded using a Cary 5000 spectrophotometer from Agilent Technologies. EDX analysis was performed with a HITACHI SU8030 microscope, utilizing cold field emissions and an ultralow‐voltage imaging resolution of 1.3 nm at 1.0 kV. FT‐IR measurements were conducted using a VERTEX70 spectrometer from Bruker.

XPS measurements were performed using a multichambered ultrahigh‐vacuum (UHV) system with a base pressure of 8 × 10^−10^ mbar. The spectrometer was equipped with a helium discharge lamp (Leybold‐Heraeus UVS10/35), a conventional Al/Mg X‐ray tube (XR50, Specs), and a hemispherical analyzer (PHOIBOS 100, Specs). The energy scale was calibrated to reproduce the binding energies of Au 4f_7/2_ (84.0 eV), Ag 3d_5/2_ (368.2 eV), and Cu 2p_3/2_ (932.6 eV). Peak fitting of XPS spectra was performed using Unifit 2018.^[^ ^34^ ^]^

All optoelectrical measurements were conducted in a probe station (Lake Shore, CRX‐6.5 K). The contact pads were contacted with W‐tips, connected to a source meter‐unit (Keithley, 2636 B).

*Intrinsic photoresponse time measurements using ASOPS.* Optical Sampling Engine (OSE) was used from Menlo Systems GmbH, featuring two femtosecond Erbium fiber lasers (*λ*_Exc_.: 1560 nm, pulse width: ≈65 fs, and *P*_Avg_.: ≈90 mW). The main repetition rate corresponds to *f*_rep_ = 100 MHz, whereas one laser was slightly detuned by Δ*f* = 100 Hz. The InSb‐Cl QD photodetector was introduced into the nitrogen‐filled sample station using a nitrogen‐filled holder from a glove box. Measurements were performed under vacuum (≈1 × 10^−5^ mbar). The temperature was controlled in the range of 10–295 K, using a Lake Shore temperature controller (model 336). The average laser pulse power was reduced by inserting fiber attenuators (3 dB, 10 dB) in the fiber‐based laser setup. The two‐pulse coincidence (2PC) dip fitting was performed by exponentially fitting both sides of the dip.

*Extrinsic photoresponse time measurements.* A square pulse laser (636 nm) was switched on and off by a Hewlett Packard 33120 arbitrary waveform generator, triggering an FSL500 (PicoQuant) driver. To measure the impulse response, a pulsed laser, with a pulse width at FWHM <500 ps at a repetition rate of 100 kHz (636 nm), controlled by a Taiko PDL M1 (PicoQuant) driver, was used. The 3 dB bandwidth of the impulse data was determined from converting the temporal response into the power spectrum obtained by the fast Fourier transform (FFT). Impedance spectroscopy was performed using a CH Instruments Electrochemical Analyzer/Workstation Model 760 E. The capacitance was calculated using the method described by Nabetl et al.^[^ ^35^ ^]^ C = L(N‐1)$\varepsilon_{0}$(1 + $\varepsilon_{r}$)(K(k))/(K(k’)). Here, *L* is the channel width (1 cm), *N* is the number of fingers (21), $\varepsilon_{0}$ is the vacuum permittivity, $\varepsilon_{r}$ is the dielectric constant of InSb QDs (15.4 for 6 nm),^[^ ^36^ ^]^ and K(k) is the complete first‐order elliptical integral with *k* = cos(*π*/2(1‐w/(w + g))) and k′ = $\sqrt{\left(1-k^{2}\right)}$. The electrode width (w) is 10 *μ*m, and the gap between the two electrodes (g) is 5 μm, resulting in a calculated capacitance of 9.47 pF.

*Statistics.* ASOPS data were first normalized and then fitted on both the right and left sides using the following equation, y = A1*$e^{-x\text{/}\tau 1}$ + A2*$e^{-x\text{/}\tau 2}$ + *y*_0_. Here, the *τ*1 and A1 represent the *τ*_In_slow_ and its corresponding coefficient, while the *τ*2 and A2 represent the *τ*_In_fast_ and its coefficient. The mean and standard deviation were calculated from the fitted results of both sides (*n* = 2). Statistical analysis was performed using the software OriginPro.

## Conflict of Interest

The authors declare no conflict of interest.

## Author Contributions

**Marcus Scheele**: directed the study and conceived the idea. **Yongju Kwon**: designed experiments and performed the syntheses, surface engineering, device fabrication, and all characterizations (XPS, ASOPS, and optoelectrical measurements); **Zhouxiaosong Zeng** and **Patrick Michel**: performed ASOPS experiments. **Fabian Strauß**: designed the extrinsic photoresponse study and performed the experiments/analysis. **Eric Juriatti**: performed XPS characterization/analysis, and **Heiko Peisert**: supervised the XPS research.

## Supporting information

Supplementary Material

## Data Availability

The data that support the findings of this study are available from the corresponding author upon reasonable request.
