# Supplementary material for: Time‐Resolved Intrinsic and Extrinsic Photoresponse of Colloidal Short‐Wavelength Infrared‐Active Indium Antimonide Quantum Dot Photodetectors
Source: Small Sci. 2025 Jul 22;5(10):2500276. doi: 10.1002/smsc.202500276 (PMC12499498; doi:10.1002/smsc.202500276)
Supplement: Supplementary file 1 — Supplementary Material [file SMSC-5-2500276-s001.pdf]

Supporting Information

# Time-Resolved Intrinsic and Extrinsic Photoresponse of Colloidal SWIR-Active Indium Antimonide Quantum Dot Photodetectors

Yongju Kwon[a], Zhouxiaosong Zeng[a], Fabian Strauß[a], Eric Juriatti[a], Patrick Michel[a], Heiko Peisert[a], and Marcus Scheele[a]*

[a] Institute of Physical and Theoretical Chemistry, University of Tübingen, Auf der Morgenstelle 15, 72076 Tübingen, Germany

## ASOPS principle

In conventional setups, both pump and probe beams operate at the same repetition rate, with a time delay between the pulses controlled by an optical delay line. In ASOPS, however, two femtosecond fiber lasers are electronically synchronized with a small difference in repetition rates.[1], [2] This results in pump and probe pulses with a continuously changing Δt. ASOPS uses two identical laser pulses with varying Δt between them, and the resulting photoresponse is measured as a function of Δt. At zero-time delay, when the pump and probe pulses coincide, the photocurrent reaches a minimum due to the sublinear power-dependent photocurrent (**Scheme S1a**). As the time delay increases from zero, the photocurrent rises exponentially from the minimum. This configuration allows the delay time to accumulate over the entire period ($1/f_{rep}$), and the dip in the photocurrent curve reflects the photodetector's ability to resolve two closely spaced pulses, showing how quickly photocarriers are generated by the first pulse response before the second pulse arrives (**Scheme S1b**).

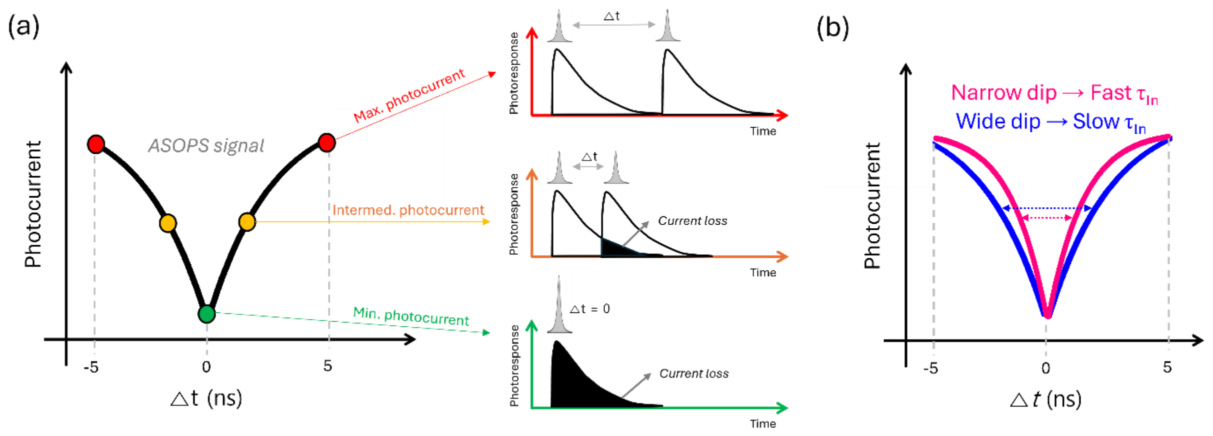


**Scheme S1.** Schematic illustration of ASOPS: (a) working principle and (b) an example of ASOPS data. The wider dip corresponds to a slow $\tau_{In}$, whereas the narrow dip reflects a fast $\tau_{In}$.

## The influencing factors on $\tau_{In}$

When photocarriers are generated in QDs, they can contribute to the photocurrent through exciton dissociation, drift, and interfacial transfer.[3] However, they may also be lost through radiative or non-radiative recombination. The overall $\tau_{In}$ comprises two main factors: the transit time ($t_{Trans.}$) and the recombination time ($\tau_{Recomb.}$) of photocarriers, as described by Eq. S1[4], [5]:

$$\frac{1}{\tau_{In}} = \frac{1}{t_{Trans.}} + \frac{1}{\tau_{Recomb.}} \cdots Eq.S1$$

To determine whether the $t_{Trans.}$ or $\tau_{Recomb.}$ dominates the $\tau_{In}$, the source-drain voltage ($V_{DS}$) can be varied, as the $t_{Trans.}$ depends on $V_{DS}$, carrier mobility ($\mu$), and channel length ($L$), according to Eq. S2.[4]

$$t_{Trans.} \sim \frac{L^2}{\mu V_{DS}} \cdots Eq.S2$$

## The influencing factors on $t_{Ex}$

The $t_{Ex}$ is influenced by three primary mechanisms: RC time ($t_{RC}$), transit time ($t_{Trans.}$), and diffusion time ($t_{Diff.}$), as described by Eq. S3.[6]

$$t_{Ex} = \sqrt{(t_{RC})^2 + (t_{Trans.})^2 + (t_{Diff.})^2} \cdots Eq.S3$$

The $t_{RC}$ is a product of 2.2, device's resistance (R) and capacitance (C), as shown in Eq. S4 for the simplified case of a plate capacitor. The $t_{Trans.}$, on the other hand, depends on the mobility (μ), channel length (L), and applied $V_{DS}$, as described by Eq. S2.[6], [7]

$$t_{RC} = 2.2 \times R \times C = 2.2 \times \frac{RA\varepsilon_r\varepsilon_0}{d} \cdots Eq.S4,$$

(A: effective area of a device, $\varepsilon_r$: dielectric constant, $\varepsilon_0$: permittivity of free space, d: material's thickness)

The diffusion time, $t_{Diff.}$, is only relevant in non-depleted regions and depends on the diffusion length and coefficient. By increasing the depletion region or carrier mobility with external bias, the diffusion zones shrink, leading to the elimination of the slow diffusion component's impact on $t_{Ex}$.

**Supporting Figures**

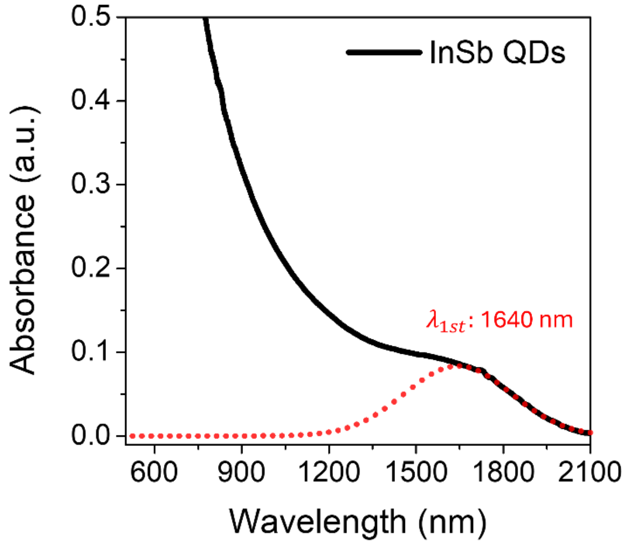


**Figure S1.** UV-vis-NIR spectrum of synthesized InSb QDs and its Gaussian fitting for the 1st excitonic absorption peak.

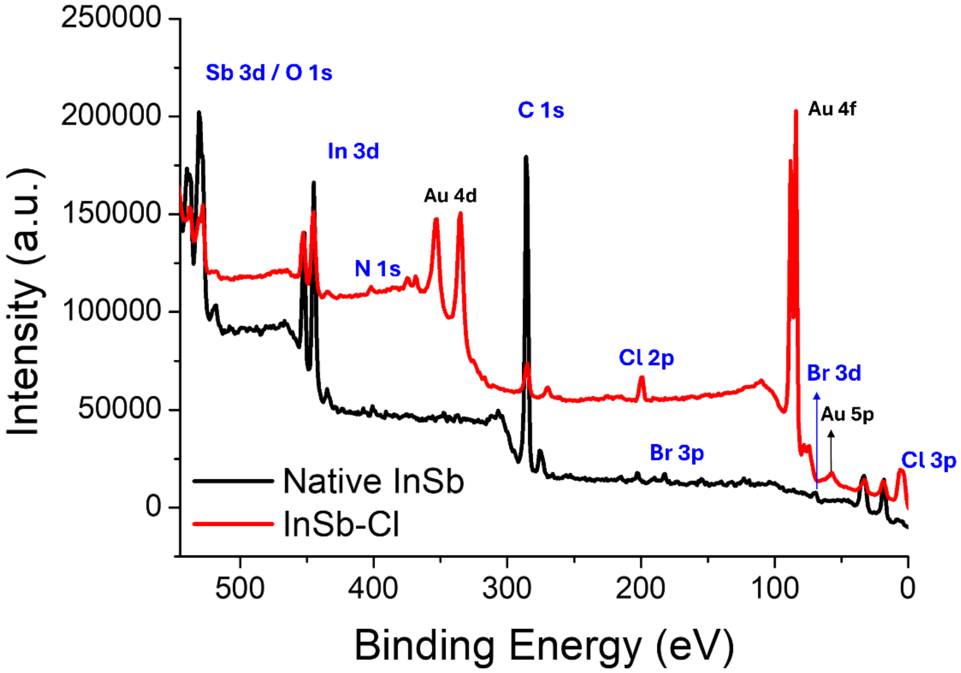


**Figure S2.** XPS survey spectra of native InSb and InSb-Cl QDs.

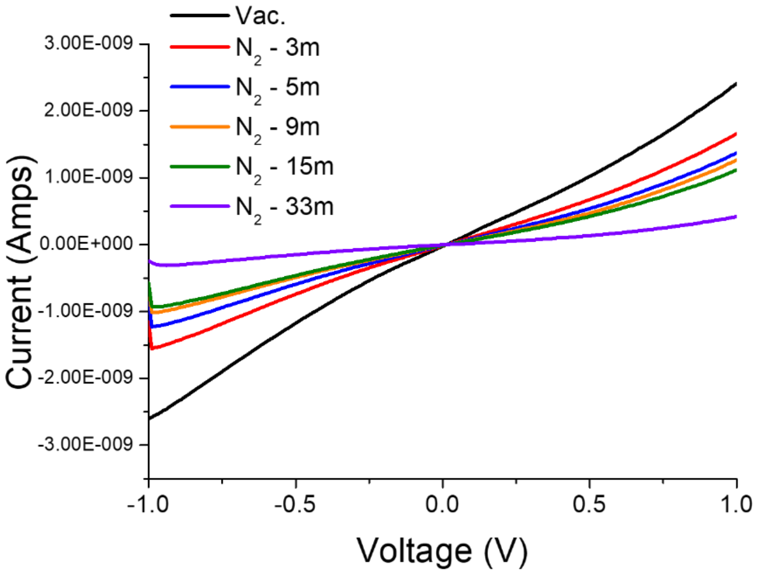


**Figure S3.** I-V curves of InSb-Cl QD photodetectors under vacuum or $N_2$ environment.

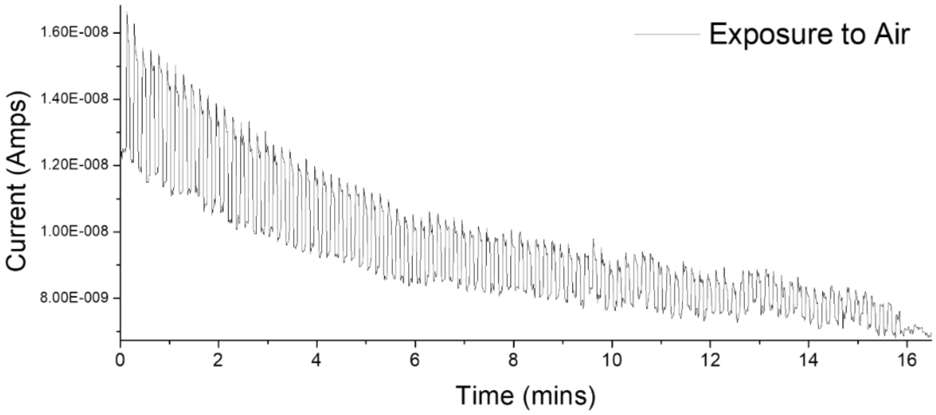


**Figure S4.** ON/OFF photoresponse characteristic of InSb-Cl QD photodetectors under air using a square pulse laser ($V_{DS}$: 1 V, $\lambda_{Exc.}$: 636 nm, $f$: 0.1 Hz, P: 55 μW).

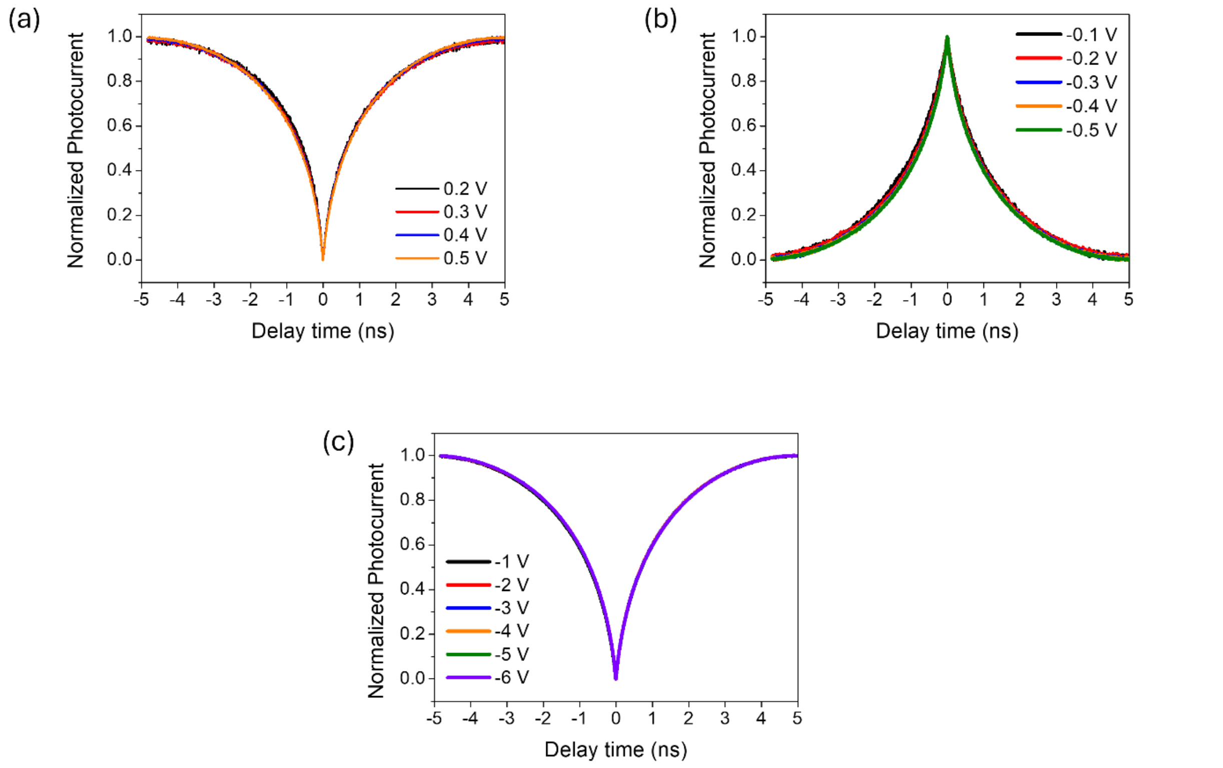


**Figure S5.** ASOPS data of InSb-Cl QD photodetectors at varying external voltages between drain and source ($V_{DS}$) (a) from 0.2 V to 0.5 V, (b) from -0.1 V to -0.5 V, and (c) from -1 V to -6 V.

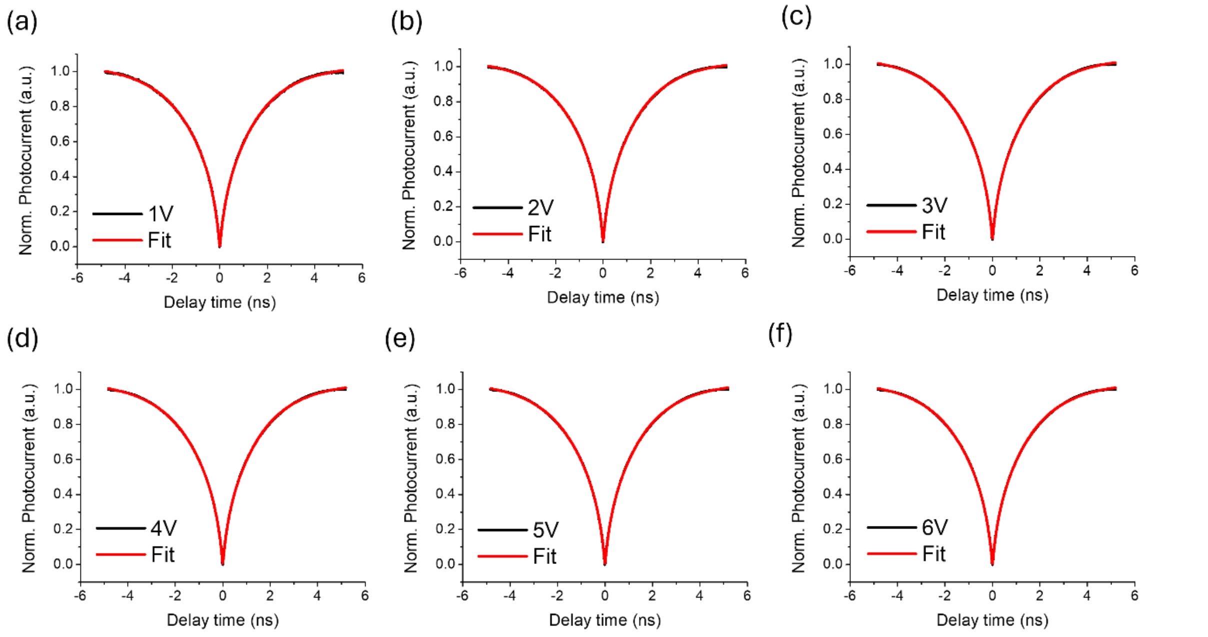


**Figure S6.** ASOPS signals and their fits using a second-order exponential decay function at $V_{DS}$ of (a) 1 V, (b) 2 V, (c) 3 V, (d) 4 V, (e) 5 V, and (f) 6 V.

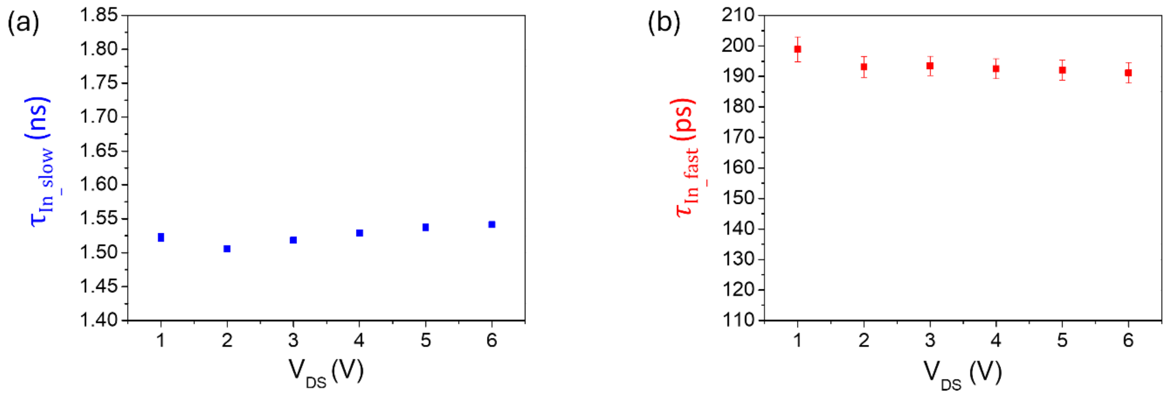


**Figure S7.** The $\tau_{In}$ for (b) $\tau_{In\_slow}$ and (c) $\tau_{In\_fast}$ components are plotted as a function of $V_{DS}$.

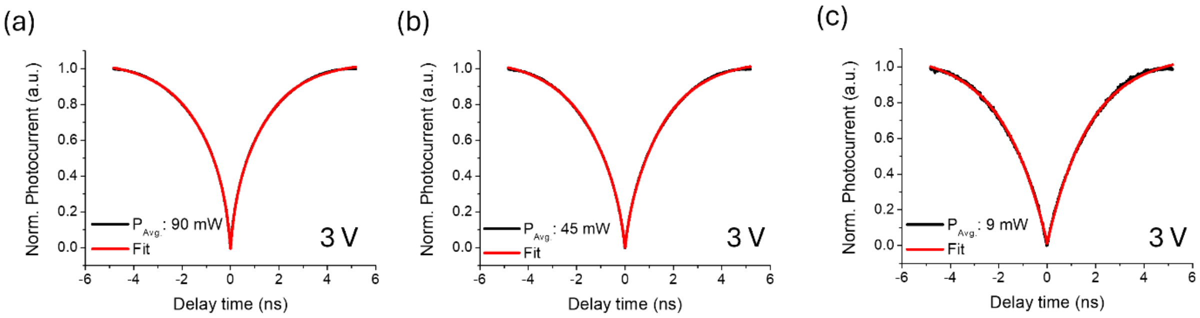


**Figure S8.** ASOPS signals and their fits using an exponential decay function under variation of the average power of the pump and probe lasers of (a) 90 mW, (b) 45 mW, and (c) 9 mW under 3 V.

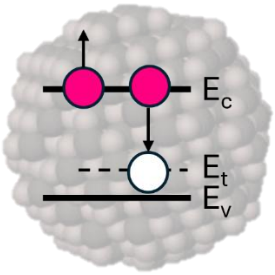


**Figure S9.** Schematic illustration of trap-assisted Auger (TAA) process. $E_C$: energy state of a conduction band edge, $E_t$: energy state of traps, $E_V$: energy state of a valence band edge.

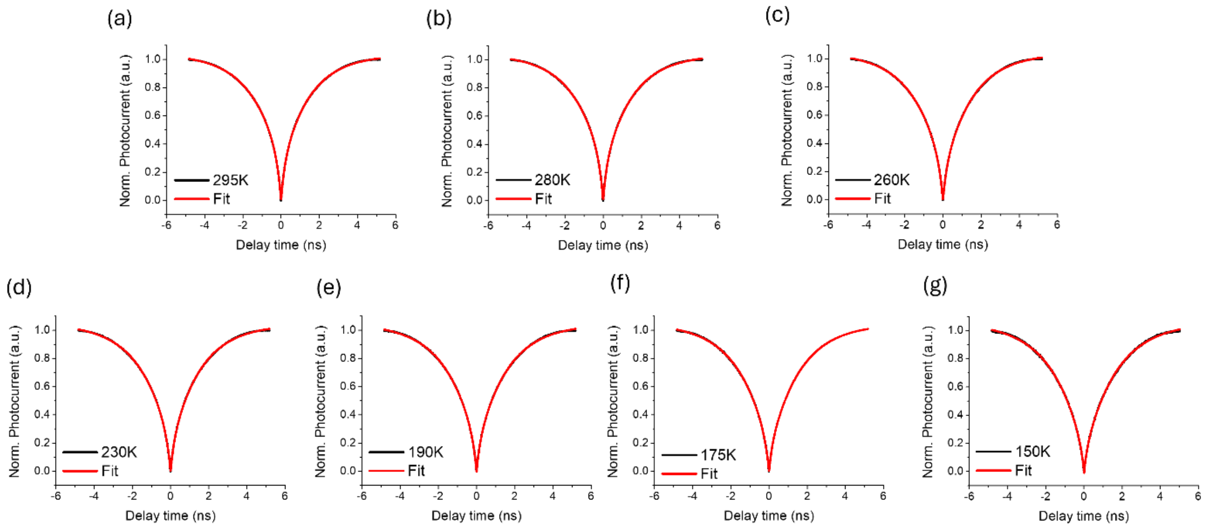


**Figure S10.** ASOPS signals and their fits using a second-order exponential decay function at different temperatures of (a) 295 K, (b) 280 K, (c) 260 K, (d) 230 K, (e) 190 K, (f) 175 K, and (g) 150 K.

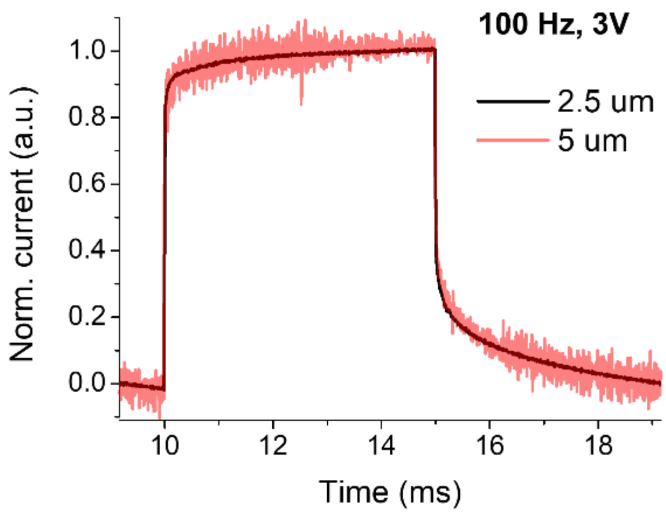


**Figure S11.** Photoresponse characteristics of InSb-Cl QD photodetectors using a square pulse laser ($V_{DS}$: 3 V, $f$: 100 Hz, $\lambda_{Exc.}$: 636 nm, P: 2 mW) for channel lengths of 2.5 µm and 5 µm.

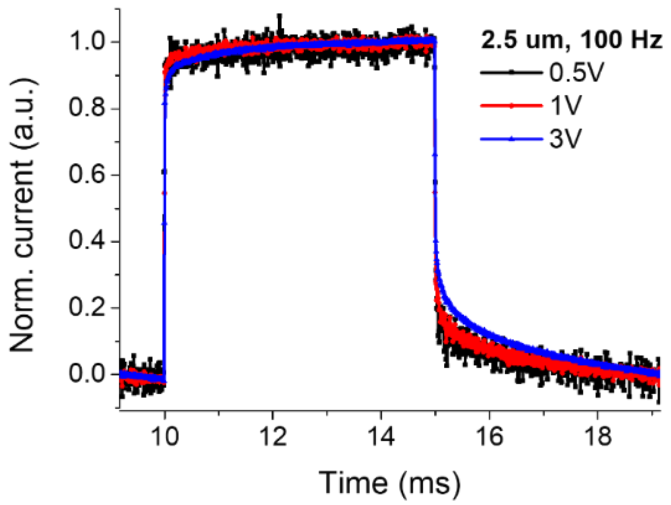


**Figure S12.** Photoresponse characteristics of InSb-Cl QD photodetectors using a square pulse laser ($f$: 100 Hz, $\lambda_{Exc.}$: 636 nm, P: 2 mW) under 0.5 V, 1 V, or 3 V for a channel length of 2.5 μm.

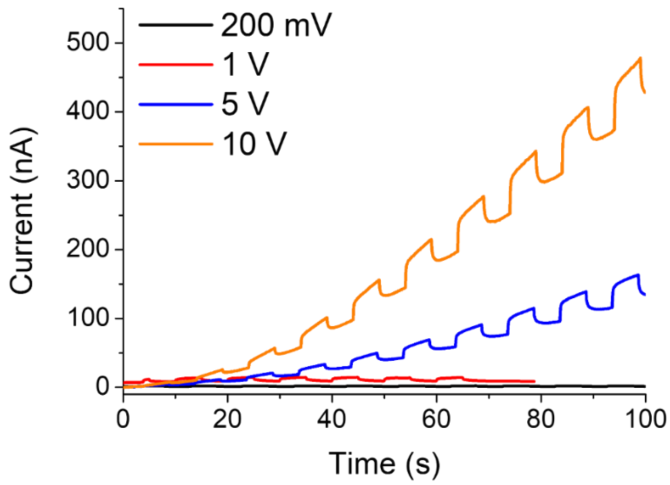


**Figure S13.** Photoresponse characteristics of InSb-Cl QD photodetectors under 200 mV, 1 V, 5 V, or 10 V ($f$: 0.1 Hz, $\lambda_{Exc.}$: 636 nm, P: 55 μW).

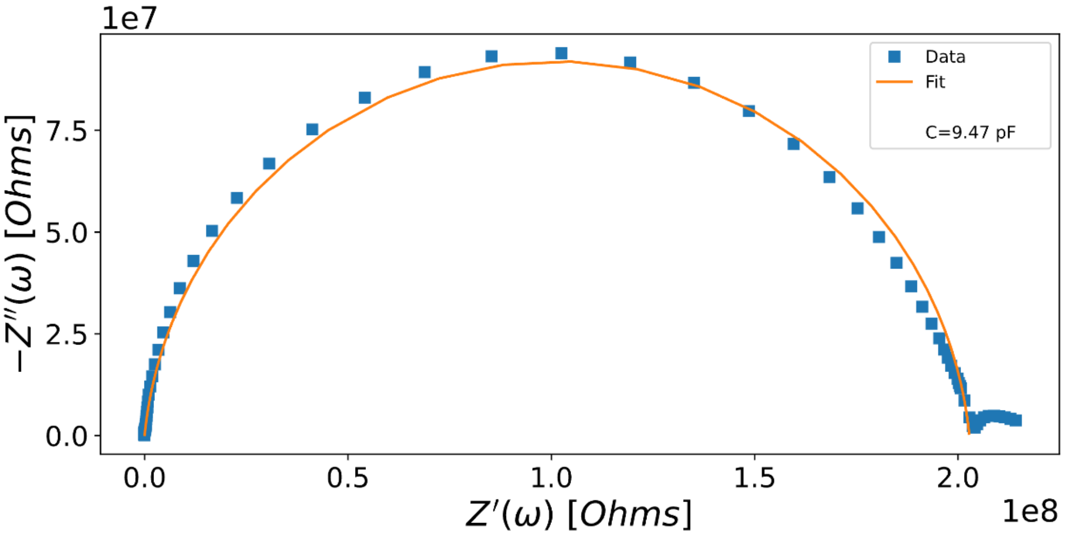


**Figure S14.** Impedance ($V_{DS}$: 0.5 V, $\lambda_{Exc.}$: 636 nm, P: 0.5 μW) data and its fitting for InSb-Cl QD photodetectors.

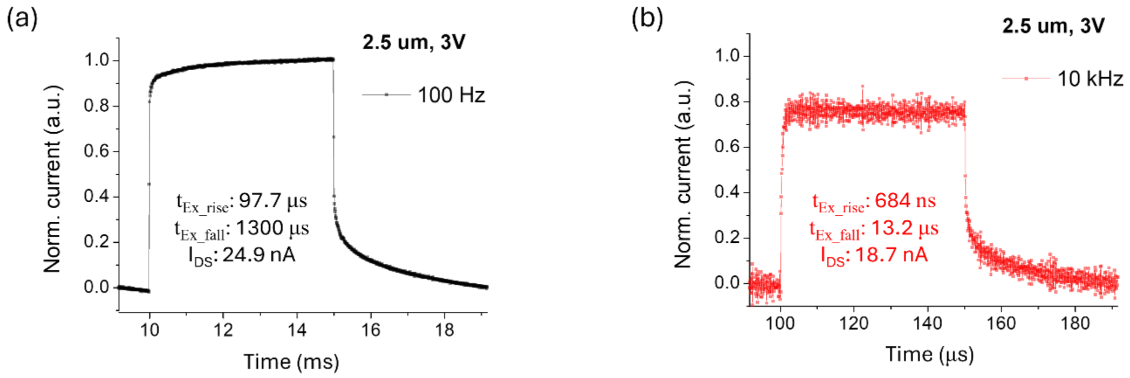


**Figure S15.** Photoresponse characteristics of InSb-Cl QD photodetectors using a square pulse laser ($V_{DS}$: 3 V, $\lambda_{Exc.}$: 636 nm, P: 2 mW) with a laser frequency of (a) 100 Hz or (b) 10 kHz for a channel length of 2.5 μm.

## Supporting Tables

**Table S1.** Comparison of the photoresponse properties in this work with prior studies.

| | Year | Sample | Device type | Wavelength for detection | Optoelectric properties | Ref. |
|---|---|---|---|---|---|---|
| **1** | 2012 | InSb-S QD | Phototransistor | 1400 nm | Electron mobilities: $1.5\times10^{-4}$ $cm^2/Vs$<br>Hole mobilities: $6\times10^{-4}$ $cm^2/Vs$ | [8] |
| **2** | 2022 | InSb-OAm QD<br>(OAm: oleylamine) | Photoconductor | 1400 nm | $t_{Ex\_rise}$: 80 ms, $t_{Ex\_fall}$: 80 ms<br>Responsivity: 0.4 mA/W<br>Detectivity: $5 \times 10^6$ Jones | [9] |
| **3** | 2023 | InSb-Br QD | Photodiode | 1200 nm | $t_{Ex\_rise}$: 550 ms, $t_{Ex\_fall}$: 800 ms<br>Responsivity: 98 mA/W | [10] |
| **4** | 2024 | InSb-S QD | Photodiode | 1200 nm | $t_{Ex\_rise}$: 200 ms, $t_{Ex\_fall}$: 200 ms<br>f3dB: 63 Hz | [11] |
| **5** | 2023 | InSb-Halide QD | Photodiode | 1200 nm | $t_{Ex\_rise}$: 7.3 μs, $t_{Ex\_fall}$: 5.4 μs<br>EQE: 75% | [12] |
| **6** | 2024 | InSb-Halide QD | Photodiode | 1400 nm | $t_{Ex}$: 5.6 μs<br>EQE: 25%<br>Detectivity: $1.4 \times 10^{11}$ Jones | [13] |
| **7** | 2024 | InSb/InP-I QD | Photodiode | 1420 nm | $t_{Ex}$: 1.65 μs – 70 ns<br>f3dB: 850 kHz (3.1 $mm^2$), 6 MHz (0.09 $mm^2$)<br>Detectivity: $4.4 \times 10^{11}$ Jones | [14] |
| **8** | 2024 | InSb/InAs-$Mn_2Se_2$ QD | Photodiode | 1370, 1520 nm | EQE: 11.4 %<br>Responsivity: 78 mA/W<br>Detectivity: $3.6 \times 10^{12}$ Jones | [15] |
| **9** | 2025 | InSb-Halide QD | Photodiode | 1380 nm | EQE: 33 %<br>Detectivity: $10^{12}$ Jones | [16] |
| **10** | 2025 | InSb/InP-I QD | Photodiode | 1200, 1380 nm | $t_{Ex\_rise}$: 83 ns, $t_{Ex\_fall}$: 95 ns<br>f3dB: 5 MHz<br>EQE: 20 %<br>Detectivity: $5.6 \times 10^{10}$ Jones | [17] |
| **11** | 2025 | InSb-ME QD<br>(ME: mercaptoethanol) | Photodiode | 1450 nm | EQE: 28 %<br>Detectivity: $1.2 \times 10^{10}$ Jones | [18] |
| **This work** | | InSb-Cl QD | Photoconductor | 1560 nm | $t_{Ex\_rise}$: 9.8 μs, $t_{Ex\_fall}$: 635 μs<br>f3dB: 5 MHz<br>$\tau_{In\_fast}$: 200 ps, $\tau_{In\_fast}$: 1.5 ns | |

**Table S2.** Energy dispersive X-ray spectroscopy (EDX) data of (a) native InSb QDs and (b) InSb-Cl QDs and their atomic ratios.

(a)

| Native InSb QDs | | |
|---|---|---|
| | **Atomic %** | **Sb=1** |
| **In** | 47.01 | 1.65 |
| **Sb** | 28.45 | 1.00 |
| **Br** | 24.54 | 0.86 |

(b)

| InSb-Cl QDs | | |
|---|---|---|
| | **Atomic%** | **Sb=1** |
| **In** | 21.51 | 1.20 |
| **Sb** | 17.95 | 1.00 |
| **Cl** | 60.19 | 3.35 |
| **Br** | 0.30 | 0.02 |

**Table S3.** The fitting parameters of (a) Br 3d / Cl 2p, (b) In 3d, and (c) Sb 3d / O 1s XPS spectra for native InSb QDs and InSb-Cl QDs.

(a)

| Br 3d / Cl 2p XPS | | | Br 3d | | Cl 2p | |
|---|---|---|---|---|---|---|
| | | | Br $3d_{5/2}$ | Br $3d_{3/2}$ | Cl $2p_{3/2}$ | Cl $2p_{1/2}$ |
| Native InSb | Binding energy (eV) | | 70.5 | 71.6 | | |
| | FWHM (eV) | Gaussian | 1.71 | 1.71 | | |
| | | Lorentzian | 0.10 | 0.10 | | |
| | Relative area (%) | | 60.0 | 40.0 | | |
| InSb-Cl | Binding energy (eV) | | | | 199.0 | 200.7 |
| | FWHM (eV) | Gaussian | | | 1.85 | 1.85 |
| | | Lorentzian | | | 0.30 | 0.10 |
| | Relative area (%) | | | | 66.7 | 33.3 |

(b)

| In 3d XPS | | | In-Sb | | In-O | | In-X (X=Br or Cl) | |
|---|---|---|---|---|---|---|---|---|
| | | | In $3d_{5/2}$ | In $3d_{3/2}$ | In $3d_{5/2}$ | In $3d_{3/2}$ | In $3d_{5/2}$ | In $3d_{3/2}$ |
| Native InSb | Binding energy (eV) | | 444.4 | 452.0 | 445.1 | 452.6 | 446.0 | 453.6 |
| | FWHM (eV) | Gaussian | 1.60 | 1.60 | 1.43 | 1.43 | 1.60 | 1.60 |
| | | Lorentzian | 0.25 | 0.25 | 0.10 | 0.10 | 0.20 | 0.20 |
| | Relative area (%) | | 9.0 | 6.0 | 26.6 | 17.7 | 24.3 | 16.3 |
| InSb-Cl | Binding energy (eV) | | 444.5 | 452.1 | 445.3 | 452.8 | 446.1 | 453.7 |
| | FWHM (eV) | Gaussian | 1.36 | 1.36 | 1.35 | 1.35 | 1.60 | 1.60 |
| | | Lorentzian | 0.30 | 0.30 | 0.30 | 0.30 | 0.40 | 0.40 |
| | Relative area (%) | | 23.8 | 16.0 | 5.2 | 3.5 | 30.9 | 20.7 |

(c)

| Sb 3d / O 1s XPS | | | Sb(-III)-In | | Sb(III)-O | | Sb(V)-O | | Oxide |
|---|---|---|---|---|---|---|---|---|---|
| | | | Sb $3d_{5/2}$ | Sb $3d_{3/2}$ | Sb $3d_{5/2}$ | Sb $3d_{3/2}$ | Sb $3d_{5/2}$ | Sb $3d_{3/2}$ | O 1s |
| Native InSb | Binding energy (eV) | | 528.6 | 538.0 | 530.1 | 539.4 | 531.3 | 540.9 | 532.7 |
| | FWHM (eV) | Gaussian | 1.52 | 1.52 | 1.80 | 1.80 | 1.79 | 1.79 | 2.22 |
| | | Lorentzian | 0.10 | 0.10 | 0.22 | 0.22 | 0.20 | 0.20 | 0.10 |
| | Relative area (%) | | 26.0 | 14.5 | 10.3 | 7.2 | 27.0 | 15.0 | - |
| InSb-Cl | Binding energy (eV) | | 528.1 | 537.5 | 529.4 | 538.8 | 531.0 | 540.4 | 532.7 |
| | FWHM (eV) | Gaussian | 1.45 | 1.45 | 1.40 | 1.40 | 1.60 | 1.60 | 2.00 |
| | | Lorentzian | 0.30 | 0.30 | 0.28 | 0.28 | 0.30 | 0.30 | 2.00 |
| | Relative area (%) | | 36.3 | 24.4 | 6.4 | 4.2 | 17.1 | 11.6 | - |

**Table S4.** The fitting parameters for the $\tau_{In\_slow}$ and $\tau_{In\_fast}$ for InSb-Cl QD photodetectors, measured at various laser powers under 3 V.

| | $P_{Avg.}$ (mW) | 90 | 45 | 9 |
|---|---|---|---|---|
| $\tau_{In\_slow}$ | \|A1\| | 0.851 (±0.001) | 0.945 (±0.001) | 1.059 (±0.001) |
| | \|$\tau$1\| (ns) | 1.555 (±0.005) | 1.656 (±0.006) | 1.810 (±0.005) |
| $\tau_{In\_fast}$ | \|A2\| | 0.189 (±0.002) | 0.115 (±0.002) | - |
| | \|$\tau$2\| (ps) | 185 (±3) | 165 (±6) | - |
| | $y_0$ | 1.042 (±0.001) | 1.056 (±0.001) | 1.074 (±0.001) |

*For the tables S4 and S5, ASOPS data on both the right and left sides were fitted using an equation, $y = A1 \times e^{(-x/\tau1)} + A2 \times e^{(-x/\tau2)} + y_0$. Here, the $\tau$1 and A1 represent the $\tau_{In\_slow}$ and its corresponding coefficient, while the $\tau$2 and A2 represent the $\tau_{In\_fast}$ and its coefficient.

**Table S5.** The fitting parameters of the $\tau_{In\_slow}$ and $\tau_{In\_fast}$ for InSb-Cl QD photodetectors, measured at various temperatures, ranging from 295 K to 150 K.

| | Temp. (K) | 295 | 280 | 260 | 230 | 190 | 175 | 150 |
|---|---|---|---|---|---|---|---|---|
| $\tau_{In\_slow}$ | \|A1\| | 0.817 (±0.001) | 0.822 (±0.001) | 0.862 (±0.001) | 0.879 (±0.001) | 0.893 (±0.001) | 0.910 (±0.001) | 0.944 (±0.002) |
| | \|$\tau$1\| (ns) | 1.497 (±0.004) | 1.510 (±0.004) | 1.544 (±0.005) | 1.559 (±0.005) | 1.585 (±0.005) | 1.608 (±0.005) | 1.673 (±0.007) |
| $\tau_{In\_fast}$ | \|A2\| | 0.215 (±0.002) | 0.210 (±0.002) | 0.179 (±0.002) | 0.166 (±0.002) | 0.150 (±0.002) | 0.141 (±0.002) | 0.114 (±0.003) |
| | \|$\tau$2\| (ps) | 190 (±3) | 189 (±3) | 187 (±3) | 183 (±4) | 176 (±4) | 166 (±5) | 159 (±7) |
| | $y_0$ | 1.035 (±0.000) | 1.037 (±0.000) | 1.042 (±0.001) | 1.044 (±0.001) | 1.045 (±0.001) | 1.048 (±0.001) | 1.056 (±0.001) |

## References


[1] A. Maier, F. Strauss, P. Kohlschreiber, C. Schedel, K. Braun, M. Scheele, "Sub-nanosecond Intrinsic Response Time of PbS Nanocrystal IR-Photodetectors" *Nano Lett* **2022**, 22, 2809. https://doi.org/10.1021/acs.nanolett.1c04938

[2] a)P. A. Elzinga, F. E. Lytle, Y. Jian, G. B. King, N. M. Laurendeau, "Pump/Probe Spectroscopy by Asynchronous Optical Sampling" *Applied Spectroscopy* **1987**, 41, 2. https://doi.org/10.1366/0003702874868025 ; b)A. Bartels, R. Cerna, C. Kistner, A. Thoma, F. Hudert, C. Janke, T. Dekorsy, "Ultrafast time-domain spectroscopy based on high-speed asynchronous optical sampling" *Rev Sci Instrum* **2007**, 78, 035107. https://doi.org/10.1063/1.2714048; c)A. Asahara, Y. Arai, T. Saito, J. Ishi-Hayase, K. Akahane, K. Minoshima, "Dual-comb-based asynchronous pump-probe measurement with an ultrawide temporal dynamic range for characterization of photo-excited InAs quantum dots" Applied Physics Express 2020, 13, 062003. https://doi.org/10.35848/1882-0786/ab8b4f

[3] M. Massicotte, P. Schmidt, F. Vialla, K. G. Schadler, A. Reserbat-Plantey, K. Watanabe, T. Taniguchi, K. J. Tielrooij, F. H. Koppens, "Picosecond photoresponse in van der Waals heterostructures" *Nat Nanotechnol* **2016**, 11, 42. https://doi.org/10.1038/nnano.2015.227

[4] A. Maier, F. Strauss, P. Kohlschreiber, C. Schedel, K. Braun, M. Scheele, "Sub-nanosecond Intrinsic Response Time of PbS Nanocrystal IR-Photodetectors" *Nano Lett* **2022**, 22, 2809. https://doi.org/10.1021/acs.nanolett.1c04938

[5] Z. Zeng, K. Braun, C. Ge, M. Eberle, C. Zhu, X. Sun, X. Yang, J. Yi, D. Liang, Y. Wang, L. Huang, Z. Luo, D. Li, A. Pan, X. Wang, "Picosecond electrical response in graphene/$MoTe_2$ heterojunction with high responsivity in the near infrared region" *Fundamental Research* **2022**, 2, 405. https://doi.org/10.1016/j.fmre.2021.09.018

[6] F. Strauss, Z. Zeng, K. Braun, M. Scheele, "Toward Gigahertz Photodetection with Transition Metal Dichalcogenides" *Acc Chem Res* **2024**, 57, 1488. https://doi.org/10.1021/acs.accounts.4c00088

[7] A. Morteza Najarian, M. Vafaie, B. Chen, F. P. García de Arquer, E. H. Sargent, "Photophysical properties of materials for high-speed photodetection" *Nature Reviews Physics* **2024**, 6, 219. https://doi.org/10.1038/s42254-024-00699-z;

[8] W. Liu, A. Y. Chang, R. D. Schaller, D. V. Talapin, "Colloidal InSb Nanocrystals" *J Am Chem Soc* **2012**, 134, 20258. https://doi.org/10.1021/ja309821j

[9] M. He, Z. Xu, S.-W. Zhang, M. Zhang, C. Wu, B. Li, J. Li, L. Wang, S. Zhao, F. Kang, G. Wei, "Colloidal InSb Quantum Dots/Organic Integrated Bulk Heterojunction for Fast and Sensitive Near-Infrared Photodetectors" *Advanced Photonics Research* **2022**, 3, 2699. https://doi.org/10.1002/adpr.202100305

[10] S. Chatterjee, K. Nemoto, B. Ghosh, H.-T. Sun, N. Shirahata, "Solution-Processed InSb Quantum Dot Photodiodes for Short-Wavelnfrared Sensing" *ACS Applied Nano Materials* **2023**, 6, 15540. https://doi.org/10.1021/acsanm.3c02221

[11] S. Chatterjee, K. Nemoto, H. T. Sun, N. Shirahata, "Rational ligand design for enhanced carrier mobility in self-powered SWIR photodiodes based on colloidal InSb quantum dots" *Nanoscale Horiz* **2024**, 9, 817. https://doi.org/10.1039/D4NH00038B

[12] Muhammad, D. Choi, D. H. Parmar, B. Rehl, Y. Zhang, O. Atan, G. Kim, P. Xia, J. M. Pina, M. Li, Y. Liu, O. Voznyy, S. Hoogland, E. H. Sargent, "Halide-Driven Synthetic Control of InSb Colloidal Quantum Dots Enables Short-Wave Infrared Photodetectors" *Adv Mater* **2023**, 35, e2306147. https://doi.org/10.1002/adma.202306147

[13] Y. Zhang, P. Xia, B. Rehl, D. H. Parmar, D. Choi, M. Imran, Y. Chen, Y. Liu, M. Vafaie, C. Li, O. Atan, J. M. Pina, W. Paritmongkol, L. Levina, O. Voznyy, S. Hoogland, E. H. Sargent, "Dicarboxylic Acid-Assisted Surface Oxide Removal and Passivation of Indium Antimonide Colloidal Quantum Dots for Short-Wave Infrared Photodetectors" *Angew Chem Int Ed Engl* **2024**, 63, e202316733. https://doi.org/10.1002/anie.202316733

[14] L. Peng, Y. Wang, Y. Ren, Z. Wang, P. Cao, G. Konstantatos, "InSb/InP Core–Shell Colloidal Quantum Dots for Sensitive and Fast Short-Wave Infrared Photodetectors" *ACS Nano* **2024**, 18, 5113. https://doi.org/10.1021/acsnano.3c12007

[15] H. Seo, H. J. Eun, A. Y. Lee, H. K. Lee, J. H. Kim, S. W. Kim, "Colloidal InSb Quantum Dots for 1500 nm SWIR Photodetector with Antioxidation of Surface" *Adv Sci (Weinh)* **2024**, 11, e2306439. https://doi.org/10.1002/advs.202306439.

[16] M. Imran, D. B. Kim, P. Xia, F. Y. Villanueva, B. Rehl, J. M. Pina, Y. Liu, Y. Zhang, O. Voznyy, E. Kumacheva, S. Hoogland, E. H. Sargent, "Control Over Metal-Halide Reactivity Enables Uniform Growth of InSb Colloidal Quantum Dots for Enhanced SWIR Light Detection" *Adv Mater* **2025**, e2420273. https://doi.org/10.1002/adma.202420273

[17] L. Peng, Y. Wang, C. Roda, A. Malla, M. Dosil, D. Mandal, G. Konstantatos, "Suppressing Colloidal Quantum Dot Multimer Fusion Leads to High-Performance InSb Infrared Photodetectors." *Adv Sci (Weinh)* **2025**, e2502775. https://doi.org/10.1002/advs.202502775

[18] Y. Zhang, M. Imran, P. Xia, Y. Chen, A. Gulsaran, Y. Liu, E. Nikbin, B. Rehl, L. Fan, F. Dinic, D. B. Kim, L. Zeng, M. Yavuz, S. Hoogland, E. H. Sargent, "Nucleophilic Covalent Ligands Enable Simultaneous Surface Reconstruction and Passivation of Colloidal InSb Quantum Dots for Stable Short-Wave Infrared Photodetectors" *Angew Chem Int Ed Engl* **2025**, e202505179. https://doi.org/10.1002/anie.202505179
